# Clinical application of serum NLRP3 on the diagnosis and prognosis of sepsis patients complicated with acute respiratory distress syndrome

**DOI:** 10.3389/fimmu.2023.1205132

**Published:** 2023-08-14

**Authors:** Qing Yang, Xiaojun Zhang, Le Luo, Jinglian Shen

**Affiliations:** ^1^ Department of Second Emergency, the Fourth Affiliated Hospital of China Medical University/China Medical University, Shenyang, Liaoning, China; ^2^ Anhui Isotech Biotechology, Ningguo, China

**Keywords:** sepsis, Acute respiratory distress syndrome (ARDS), NOD-like receptor family pyrin domain containing 3 (NLRP3), ROC (receiver operating characteristics) analysis, clinics

## Abstract

**Introduction:**

Acute respiratory distress syndrome (ARDS) is a common complication of sepsis, which significantly increases the mortality rate. This work explored the diagnostic value of serum NOD-like receptor family pyrin domain containing 3 (NLRP3) concentration in patients with sepsis for ARDS, and the predictive value of serum NLRP3 concentration at the time of diagnosis for death 28 days after treatment.

**Methods:**

A total of 150 sepsis patients were included in this study, including age-matched two groups of patients, 75 patients with ARDS and 75 patients without ARDS. In addition, 60 age-matched healthy patients with physical examination were recruited in this study. Serum NLRP3 concentration was determined by enzyme-linked immunosorbent assay (ELISA). The diagnostic values of serum NLRP3 concentration for ARDS in sepsis patients were evaluated by receiver operating characteristics (ROC) analysis. Correlation of serum NLRP3 with APACHE II score and SOFA were performed by Spearman correlation analysis.

**Results:**

Pulmonary infection, APACHE II score and serum NLRP3 concentration were risk factors for patients with sepsis complicated with ARDS. ROC curve results showed that the specificity of serum NLRP3 concentration was 74.67%, the sensitivity was 76.00%, and the area under the curve (AUC) was 0.82 (p<0.001). APACHE II score and SOFA were significantly positively correlated with serum NLRP3 concentration. Baseline serum NLRP3 levels had significant predictive value for 28-day mortality in sepsis patients complicated with ARDS.

**Conclusion:**

Serum NLRP3 concentration has clinical value in the diagnosis of sepsis complicated with ARDS.

## 1 Introduction

Sepsis is an emergency condition caused by infection which might lead to acute respiratory distress syndrome (ARDS) (1). Sepsis patients who develop ARDS have a higher mortality rate compared to those without ARDS (2). It was reported that the mortality rate in sepsis patients with ARDS was 46%, compared to 25% in sepsis patients without ARDS (3).

ARDS is characterized by severe inflammation and damage to the lung tissue, and its pathogenesis is complex and multifactorial (4). The development of ARDS in sepsis patients is thought to be due to an exaggerated immune response that leads to inflammation and damage to the lung tissue (5). Early recognition and treatment of sepsis are critical in preventing the development of ARDS. The Surviving Sepsis Campaign guidelines recommend early recognition of sepsis (6). Treatment for sepsis patients with ARDS typically involves supportive care and lung-protective ventilation strategies (7). These strategies, such as low tidal volume ventilation, have been shown to improve outcomes in sepsis patients with ARDS (8). Other therapies that have been investigated for sepsis-associated ARDS include corticosteroids and extracorporeal membrane oxygenation (ECMO) (9, 10).

NOD-like receptor family pyrin domain containing 3 (NLRP3) belongs to the NLR family of pattern recognition receptors (PRRs) (11). NLRP3 plays a critical role in the innate immune response, which senses danger signals and initiates an immune response (12). NLRP3 is involved in the formation of the NLRP3 inflammasome (13). Dysregulation of NLRP3 and the NLRP3 inflammasome has been found in the pathogenesis of various diseases, including autoimmune diseases and neurodegenerative diseases (14). Notably, previous studies have shown that dysregulation of NLRP3 is involved in the pathogenesis of sepsis. Previous studies have found that NLRP3 inflammasome participates in the pathogenesis of sepsis-induced kidney failure (15) and sepsis-induced liver injury (16). In addition, SIRT1/NF-κB/NLRP3 pathway has been shown to participate in acute lung injury-like diseases by regulating pyroptosis (17). TXNIP/NLRP3 and NF-κB have also been shown to regulate lipopolysaccharide-induced ARDS (18). Currently, there are no specific biomarkers available that can accurately and reliably diagnose ARDS in septic patients. While certain biomarkers, such as plasma cytokine levels and surfactant protein D, have been studied, their diagnostic utility is limited due to variability and lack of specificity (19). Therefore, the present study aimed to study the diagnostic value of serum NLRP3 concentration for ARDS in sepsis patients, and the predictive value of serum NLRP3 concentration at the time of diagnosis for death 28 days after treatment.

## 2 Methods

### 2.1 Participants

A total of 150 sepsis patients were included in this study, including age-matched two groups of patients, 75 patients with ARDS and 75 patients without ARDS. In addition, 60 age-matched healthy patients with physical examination were recruited in this study. Our study is a retrospective study, and researchers who performed data collection (collecting clinical characteristics of patients, analyzing Serum NLRP3 concentration, following up clinical outcomes of patients, etc.) were blind to the grouping of patients in the two groups. The study was approved by the ethics committee of the Fourth Affiliated Hospital of China Medical University/China Medical University, and all the participants signed informed written consent.

Inclusion criteria: meet the diagnostic criteria for sepsis and ARDS; age ≥ 18 years; complete clinical data; informed consent of patients and their families.

Exclusion criteria: accompanied by other lung diseases, including bronchial asthma, active tuberculosis, bronchiectasis, pulmonary cystic fibrosis, lung abscess, and interstitial lung disease, etc.; combined with immune system diseases, mental illness, severe heart, brain, liver and kidney insufficiency, and blood system diseases; died within 24 hours of admission; suffering from malignant tumors.

Sepsis was diagnosed according to the “Guidelines for the Emergency Treatment of Sepsis and Septic Shock in China (2018)”.

2012 Berlin diagnostic criteria: 1) Acute onset, with clear risk factors; 2) PaO2/FiO2 (P/F) ratio ≤ 300, oxygenation index ≤ 300 mmHg (1 mmHg = 0.1333 kPa); 3) Bilateral chest infiltrates on X-ray 4) Respiratory failure.

### 2.2 Serum concentration of NLRP3

Fasting venous blood (3 mL) were collected from patients on the morning of the second day after admission and from healthy subjects on the day of physical examination. The serum was separated by centrifugation at 1000 g/min for 10 min in anticoagulant tubes, and were stored at -80°C. They were stored and assessed as a batch. Serum samples were tested triplicate. The concentration of NLRP3 in the serum was determined by enzyme-linked immunosorbent assay (ELISA). The kit was purchased from Wuhan Fine Biotech (EH4202), with a detection range of 0.781-50 ng/mL and a sensitivity of 0.469 ng/mL.

### 2.3 Patient information

General demographic information (age, gender) and clinical data of patients were collected, including basic medical history, whether septic shock occurred and multiple scores, etc. The Acute Physiology and Chronic Health Evaluation II (APACHE II) score is scored from three aspects: acute physiology, age, and chronic health. The acute physiology score was calculated based on the worse value of clinical indicators (body temperature, heart rate, respiration, oxygenation, mean arterial pressure, etc.) within 24 hours of admission. Chronic health is scored. The Sequential Organ Failure Assessment (SOFA) score is calculated. SOFA is a scoring system used to assess the extent of organ dysfunction in critically ill patients. It is widely employed in intensive care units (ICUs) to monitor and predict patient outcomes. The SOFA score provides a quantifiable measure of organ dysfunction by evaluating the function of six different organ systems: respiratory, cardiovascular, hepatic, coagulation, renal, and neurological. The scoring system assigns points based on the severity of dysfunction in each organ system. Higher scores indicate more severe dysfunction and are associated with poorer outcomes. The maximum score for each organ system is four, with a total possible score of 24.

### 2.4 Clinical treatment plan and prognosis judgment

On the basis of treating the primary disease, the treatment plan recommended by the “China Sepsis/Septic Shock Emergency Treatment Guidelines (2018)” formulated by the Chinese Medical Association Intensive Care Medicine Branch was adopted. Immediately after admission, the patient was given broad-spectrum antibiotics for anti-infection, gastric mucosal protection to prevent stress ulcers, pulmonary hygiene, nutritional support, maintenance of electrolyte acid-base balance, mechanical ventilation, and possible treatment for the primary disease. At the same time, the patient was given subcutaneous 5000 IU low-molecular-weight heparin sodium, 12 hours each time, for a total of 7 days. Follow-up was conducted for 28 days, and the survival of patients with sepsis complicated with ARDS was counted.

### 2.5 Statistical analysis

The data are shown as mean ± SD or n (percentage). The comparisons of data were done by Mann-Whitney test, Unpaired t test with Welch’s correction or Fisher’s exact test. The comparisons of data from multiple groups were done by Brown-Forsythe ANOVA test followed by Dunnett’s T3 multiple comparisons test. Correlation of serum NLRP3 with APACHE II score and SOFA were performed by Spearman correlation analysis. ROC analysis (receiver operating characteristics) was used to determine the diagnostic values of serum NLRP3 concentration. *p < 0.05, **p < 0.01, ***p < 0.001.

## 3 Results

### 3.1 Clinical characteristics of sepsis participants complicated with ARDS or without ARDS

**Table 1** is a comparison of the basic conditions of 75 sepsis patients with ARDS (SARDS) and 75 sepsis patients without ARDS (SNARDS) at the time of admission. Significant differences were observed between the two groups in pulmonary infection (p<0.001), mechanical ventilation (p=0.005), septic shock(p=0.022), APACHE II score (p<0.001), SOFA (p=0.002), and serum NLRP3 (p<0.001). In addition, the average age of 60 included healthy patients was 61.17 ± 9.62, including 33 males and 27 females, all of whom had no underlying diseases. With concurrent ARDS (0=no, 1=yes) as the dependent variable, age, gender, lung infection, mechanical ventilation, septic shock, APACHE II score, SOFA score, diabetes mellitus, hypertension, hyperlipidemia, coronary heart disease, smoking history, and serum NLRP3 concentration were independent variables, and irrelevant items were gradually excluded (p>0.05). The results showed that pulmonary infection (p=0.003), APACHE II score (p<0.001) and serum NLRP3 concentration (p=0.007) were independent risk factors for sepsis patients complicated with ARDS (**Table 2**).

**Table 1:** Clinical characteristics of sepsis patients complicated with acute respiratory distress syndrome (SARDS) or not (SNARDS).

Factors	SNARDS (n=75)	SARDS (n=75)	p value
Age (years)	60.24 ± 10.01	62.92 ± 12.43	0.148
Gender			
Male	39 (52%)	46 (61.3%)	0.323
Female	36 (48%)	29 (38.7%)	
Pulmonary infection			
Yes	19 (25.3%)	42 (56%)	< 0.001
No	56 (74.7%)	33 (44%)	
Mechanical ventilate			
Yes	26 (34.7%)	44 (58.7%)	0.005
No	49 (65.3%)	31 (41.3%)	
Sepsis shock			
Yes	33 (44%)	48 (64%)	0.022
No	42 (56%)	27 (36%)	
Complicated diabetes mellitus			
Yes	23 (30.7%)	29 (38.7%)	0.391
No	52 (69.3%)	46 (61.3%)	
Complicated hypertension			
Yes	21 (28%)	26 (34.7%)	0.482
No	54 (72%)	49 (65.3%)	
Complicated hyperlipidemia			
Yes	25 (33.3%)	31 (41.3%)	0.399
No	50 (66.7%)	44 (58.7%)	
Complicated coronary heart disease			
Yes	14 (18.7%)	17 (22.7%)	0.687
No	61 (81.3%)	58 (77.3%)	
Smoke			
Yes	22 (29.3%)	30 (40%)	0.229
No	53 (70.7%)	45 (60%)	
APACHE II score	16.56 ± 6.78	24.84 ± 9.96	< 0.001
SOFA	6.84 ± 3.21	9.49 ± 5.44	0.002
Serum NLRP3 (ng/mL)	3.35 ± 1.24	5.35 ± 1.74	< 0.001

The data are shown as mean ± SD or n (percentage). The comparisons of data were done by Mann-Whitney test, Unpaired t test with Welch’s correction or Fisher’s exact test.

APACHE II, Acute Physiology and Chronic Health Evaluation II; SOFA, Sequential Organ Failure Assessment; NLRP3, NOD-like receptor thermal protein domain associated protein 3.

**Table 2:** Multivariate logistic analysis for acute respiratory distress syndrome in sepsis patients.

	OR	95% CI	p value
Pulmonary infection	1.655	1.498 to 1.812	0.003
APACHE II score	1.894	1.503 to 2.199	< 0.001
Serum NLRP3	1.267	1.114 to 1.408	0.007

### 3.2 Diagnostic values of serum NLRP3 concentration for ARDS in sepsis patients

**Figure 1:**
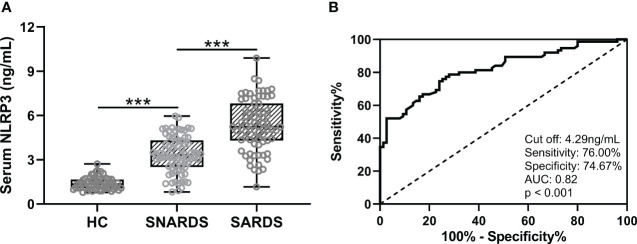
**(A)**, Comparisons of serum NLRP3 among sepsis patients complicated with ARDS (SARDS, n = 75), or not (SNARDS, n = 75) and healthy control (n = 60). ***p < 0.001 from Brown-Forsythe ANOVA test followed by Dunnett’s T3 multiple comparisons test. **(B)**, ROC analysis of diagnostic values of serum NLRP3 for ARDS in sepsis patients.

We first compared the differences in serum NLRP3 concentrations in 75 sepsis patients with ARDS (SARDS), 75 sepsis patients without ARDS (SNARDS), and 60 age-matched healthy controls. It could be seen that NLRP3 was significantly upregulated in sepsis patients than that of controls, and the patients with ARDS were higher than those without ARDS (**Figure 1A**). ROC analysis showed the diagnostic values of serum NLRP3 concentration for ARDS in sepsis patients (**Figure 1B**). The ROC curve results showed that the cut off value was 4.29 ng/mL, the sensitivity was 76.00%, the specificity was 74.67%, and AUC was 0.82 (p<0.001).

### 3.3 Correlation of serum NLRP3 concentrations with risk factors for sepsis patients complicated with ARDS

**Figure 2:**
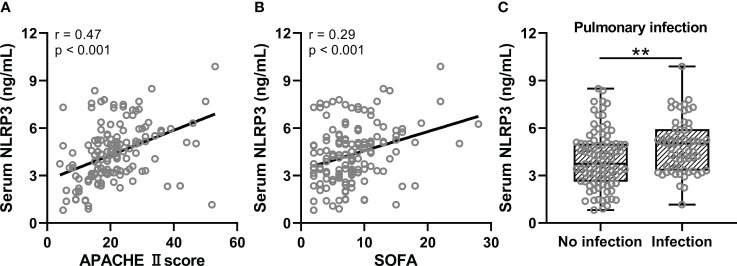
Spearman correlation analysis of serum NLRP3 with APACHE II score **(A)** and SOFA **(B)** in sepsis patients (n = 150). **(C)**, Comparisons of serum NLRP3 between the sepsis patients with pulmonary infection (n = 61) or not (n = 89). **p < 0.01.

We then analyzed the correlation of serum NLRP3 concentrations with sepsis severity and concurrent ARDS risk factors in all sepsis patients (n = 150). The results showed that APACHE II score (**Figure 2A**, r=0.47, p<0.001) and SOFA (**Figure 2B**, r=0.29, p<0.001) were significantly positively correlated with serum NLRP3 concentration. In addition, serum NLRP3 concentrations of sepsis patients with pulmonary infection (pneumonia with or without ARDS) were significantly elevated (**Figure 2C**, p<0.01).

### 3.4 The predictive value of serum NLRP3 concentration on the prognosis of sepsis

**Figure 3:**
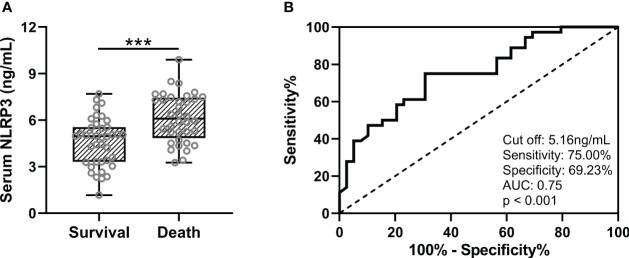
**(A)**, Comparisons of serum NLRP3 between survival (n = 39) and death (n = 36) in sepsis patients complicated with ARDS after 28 days of follow-up. **(B)**, ROC analysis of predictive values of serum NLRP3 for 28 days of death in sepsis patients complicated with ARDS. ***p < 0.001.

Subsequently, we followed up 75 patients with sepsis and ARDS for 28 days. During the follow-up, 36 cases died and 39 cases survived. We compared serum NLRP3 levels at baseline between the two groups, and analyzed the predictive value of baseline serum NLRP3 levels for 28-day mortality. It was found that patients with sepsis and ARDS had higher serum NLRP3 concentrations at baseline in patients who died at 28-day follow-up (**Figure 3A**, p<0.001), and the baseline serum NLRP3 levels had a significant predictive value for 28-day death (**Figure 3B**, p<0.001). The ROC curve results revealed that when the cut off value was 5.16 ng/mL, the sensitivity of serum NLRP3 concentration was 75.00%, the specificity was 69.23%, and the AUC was 0.75. In order to compare the changes of serum NLRP3 concentration in patients before and after treatment, we supplemented the concentration of NLRP3 in serum after treatment with retained blood samples of patients after 7 days of treatment. The results showed that after treatment in 75 sepsis patients with ARDS (SARDS) and 75 sepsis patients without ARDS (SNARDS), the concentration of NLRP3 in serum was significantly reduced (**Figure S1**). We also compared serum NLRP3 concentrations at admission between dead and alive subgroups, and the results are shown in **Figure S2**. It can be seen that the concentration of serum NLRP3 in the dead patients was higher on admission, but there was no significant difference. The area under the ROC analysis curve was 0.67, but it was not significant either (**Figure S2**).

## 4 Discussion

Early diagnosis and accurate prognosis of sepsis complicated with ARDS are crucial to improving patient outcomes (1, 20). In recent years, studies have focused on identifying reliable biomarkers for sepsis diagnosis and prognosis (21). However, the diagnosis and management of sepsis patients complicated with ARDS could be challenging (22). Currently, there is a need for biomarkers that could accurately diagnose and predict outcomes in these patients (23).

NLRP3 is a cytoplasmic protein involved in the function of the inflammasome, which participates in the innate immune response (24). In sepsis, the inflammasome is activated, which causes the release of multiple cytokines (25). Accumulating studies have suggested that serum levels of NLRP3 may have potential clinical applications in sepsis patient diagnosis (26). One study demonstrated that activated NLRP3 inflammasomes in alveolar macrophages promote the lung inflammation, which suggests a potential role for NLRP3 in the development of ARDS (27). It has been reported that inflammatory signals could activate autophagy, which in turn restricts the production of IL-1β by selectively targeting ubiquitinated inflammasomes, including NLRP3, for degradation. This suggests a potential regulatory role for autophagy in NLRP3-mediated inflammation and ARDS (28). Marivee Borges-Rodriguez el at. demonstrated that inhibition of the NLRP3 inflammasome attenuated sepsis-induced platelet activation and prevented thrombosis, suggesting a potential role for NLRP3 in the development of coagulation disorders in sepsis patients with ARDS (29). Li D et al. demonstrated that NLRP3 inflammasome activation contributes to lung injury and cytokine release in mice with ARDS, suggesting a potential therapeutic target for ARDS in sepsis patients (30). In summary, studies have shown that NLRP3 activation plays a critical role in the pathogenesis of sepsis-induced ARDS. Activation of the NLRP3 inflammasome has been implicated in the pathogenesis of lung inflammation, injury, fibrosis, and coagulation disorders in sepsis patients with ARDS. Inhibition of the NLRP3 inflammasome and activation of autophagy have shown promise as potential therapeutic targets for ARDS in sepsis patients. Although serum NLRP3 has potential for diagnosing and predicting prognosis in sepsis, its clinical application is still in its infancy, and it remains uncertain whether it will eventually be adopted as a standard biomarker in clinical practice.

Our study demonstrated that the serum NLRP3 concentration might be a useful diagnostic and prognostic biomarker for sepsis patients complicated with ARDS. In this study, the best critical value of NLRP3 diagnosis of ARDS in sepsis patients is 4.29 ng/ml, the sensitivity is 76.00%, and the specificity is 74.67%, which suggests that the abnormal elevation of NLRP3 level has certain warning significance to ARDS in sepsis patients. Moreover, high serum NLRP3 levels at admission are associated with increased mortality, severity of sepsis, and risk factors for ARDS, which has important reference significance for clinical diagnosis and prognosis evaluation. Our data suggest that serum NLRP3 levels are significantly higher in sepsis patients complicated with ARDS and may be a potential biomarker for sepsis diagnosis and prognosis. Our findings provide promising insights into the potential utility of serum NLRP3 in the diagnosis and prognosis of sepsis complicated with ARDS. However, future studies should be done to investigate the mechanisms underlying the association between serum NLRP3 levels and sepsis complicated with ARDS.

In addition, it is important to note that this study have some limitations, including small sample sizes and retrospective designs. A sample size of 75 patients in each group might limit the statistical power and may not be representative of the larger population. Our results suggested that the concentration of serum NLRP3 in the dead patients was higher on admission, but there was no significant difference. This may be due to the bias caused by the low sample size. Also, a 28-day follow-up period might not be sufficient to determine long-term outcomes, especially for a severe condition like sepsis with ARDS. It is worth noting that more of the ARDS patients are noted to be on mechanical ventilation which could in itself cause an inflammatory response. While it can provide critical support, there are several factors that can contribute to the development of more serious complications in patients receiving mechanical ventilation. Mechanical ventilation itself can lead to lung injury, known as ventilator-associated lung injury (VALI). Factors such as high tidal volumes, high airway pressures, and excessive positive end-expiratory pressure (PEEP) can contribute to VALI. Mechanical ventilation can exert Excessive pressure on the lungs, leading to barotrauma and volutrauma. Patients on mechanical ventilation are at an increased risk of developing ventilator-associated pneumonia (VAP). The presence of an artificial airway provides a pathway for bacteria to enter the lungs. Mechanical ventilation itself is also a factor affecting patient prognosis. Future studies were necessary for the determination of the clinical utility of serum NLRP3 in the diagnosis of sepsis patients with ARDS.

## 5 Conclusion

In conclusion, our data suggest that serum NLRP3 might be a useful diagnostic and prognostic biomarker for sepsis patients complicated with ARDS. High serum NLRP3 levels were associated with increased mortality rates, poor clinical outcomes, and lung injury severity in sepsis patients with ARDS. Therefore, targeting the NLRP3 inflammasome pathway might be a potential therapeutic strategy for sepsis patients with ARDS.

## Data availability statement

The raw data supporting the conclusions of this article will be made available by the authors, without undue reservation.

## Ethics statement

The studies involving human participants were reviewed and approved by the Fourth Affiliated Hospital of China Medical University/China Medical University. The patients/participants provided their written informed consent to participate in this study. Written informed consent was obtained from the individual(s) for the publication of any potentially identifiable images or data included in this article.

## Author contributions

Data curation: QY, XZ, LL and JS. Project administration: JL. Supervision: JS. Writing - original draft: QY and JS. Writing - review and editing: QY, XZ, LL and JS.

## References

[1] Salomao R Ferreira BL Salomao MC Santos SS Azevedo LCP Brunialti MKC. Sepsis: evolving concepts and challenges. Braz J Med Biol Res (2019) 52:e8595. doi: 10.1590/1414-431x20198595 30994733 PMC6472937

[2] Xu N Guo H Li X Zhao Q Li J. A five-genes based diagnostic signature for sepsis-induced ARDS. Pathol Oncol Res (2021) 27:580801. doi: 10.3389/pore.2021.580801 34393665 PMC8357742

[3] Villar J Blanco J Anon JM Santos-Bouza A Blanch L Ambros A. The ALIEN study: incidence and outcome of acute respiratory distress syndrome in the era of lung protective ventilation. Intensive Care Med (2011) 37:1932–41. doi: 10.1007/s00134-011-2380-4 21997128

[4] Huppert LA Matthay MA Ware LB. Pathogenesis of acute respiratory distress syndrome. Semin Respir Crit Care Med (2019) 40:31–9. doi: 10.1055/s-0039-1683996 PMC7060969 31060086

[5] Meyer NJ Gattinoni L Calfee CS. Acute respiratory distress syndrome. Lancet (2021) 398:622–37. doi: 10.1016/S0140-6736(21)00439-6 PMC8248927 34217425

[6] Rhodes A Evans LE Alhazzani W Levy MM Antonelli M Ferrer R. Surviving sepsis campaign: international guidelines for management of sepsis and septic shock: 2016. Crit Care Med (2017) 45:486–552. doi: 10.1097/CCM.0000000000002255 28098591

[7] Yildirim F Karaman I Kaya A. Current situation in ARDS in the light of recent studies: Classification, epidemiology and pharmacotherapeutics. Tuberk Toraks (2021) 69:535–46. doi: 10.5578/tt.20219611 34957747

[8] Fan E Del Sorbo L Goligher EC Hodgson CL Munshi L Walkey AJ. An official American thoracic society/European society of intensive care medicine/society of critical care medicine clinical practice guideline: mechanical ventilation in adult patients with acute respiratory distress syndrome. Am J Respir Crit Care Med (2017) 195:1253–63. doi: 10.1164/rccm.201703-0548ST 28459336

[9] Gibbison B Lopez-Lopez JA Higgins JP Miller T Angelini GD Lightman SL. Corticosteroids in septic shock: a systematic review and network meta-analysis. Crit Care (2017) 21:78. doi: 10.1186/s13054-017-1659-4 28351429 PMC5371269

[10] Banavasi H Nguyen P Osman H Soubani AO. Management of ARDS - what works and what does not. Am J Med Sci (2021) 362:13–23. doi: 10.1016/j.amjms.2020.12.019 34090669 PMC7997862

[11] Shao BZ Xu ZQ Han BZ Su DF Liu C. NLRP3 inflammasome and its inhibitors: a review. Front Pharmacol (2015) 6:262. doi: 10.3389/fphar.2015.00262 26594174 PMC4633676

[12] Kelley N Jeltema D Duan Y He Y. The NLRP3 inflammasome: an overview of mechanisms of activation and regulation. Int J Mol Sci (2019) 20:3328. doi: 10.3390/ijms20133328 31284572 PMC6651423

[13] Mangan MSJ Olhava EJ Roush WR Seidel HM Glick GD Latz E. Targeting the NLRP3 inflammasome in inflammatory diseases. Nat Rev Drug Discovery (2018) 17:588–606. doi: 10.1038/nrd.2018.97 30026524

[14] Wang L Hauenstein AV. The NLRP3 inflammasome: Mechanism of action, role in disease and therapies. Mol Aspects Med (2020) 76:100889. doi: 10.1016/j.mam.2020.100889 32859386

[15] He L Peng X Zhu J Chen X Liu H Tang C. Mangiferin attenuate sepsis-induced acute kidney injury via antioxidant and anti-inflammatory effects. Am J Nephrol (2014) 40:441–50. doi: 10.1159/000369220 25427663

[16] Zou L Li C Chen X Yu F Huang Q Chen L. The anti-inflammatory effects of cinnamyl alcohol on sepsis-induced mice via the NLRP3 inflammasome pathway. Ann Transl Med (2022) 10:48. doi: 10.21037/atm-21-6130 35282107 PMC8848357

[17] Zhang Y Zhang H Li S Huang K Jiang L Wang Y. Metformin alleviates LPS-induced acute lung injury by regulating the SIRT1/NF-kappaB/NLRP3 pathway and inhibiting endothelial cell pyroptosis. Front Pharmacol (2022) 13:801337. doi: 10.3389/fphar.2022.801337 35910360 PMC9334876

[18] Fang XZ Ge YL Chen ZY Shu HQ Yang YY Yu Y. NecroX-5 alleviate lipopolysaccharide-induced acute respiratory distress syndrome by inhibiting TXNIP/NLRP3 and NF-kappaB. Int Immunopharmacol (2020) 81:106257. doi: 10.1016/j.intimp.2020.106257 32044659

[19] Saguil A Fargo MV. Acute respiratory distress syndrome: diagnosis and management. Am Fam Physician (2020) 101:730–8. 32538594

[20] Faix JD. Biomarkers of sepsis. Crit Rev Clin Lab Sci (2013) 50:23–36. doi: 10.3109/10408363.2013.764490 23480440 PMC3613962

[21] Napolitano LM. Sepsis 2018: definitions and guideline changes. Surg Infect (Larchmt) (2018) 19:117–25. doi: 10.1089/sur.2017.278 29447109

[22] Rello J Valenzuela-Sanchez F Ruiz-Rodriguez M Moyano S. Sepsis: a review of advances in management. Adv Ther (2017) 34:2393–411. doi: 10.1007/s12325-017-0622-8 PMC5702377 29022217

[23] Purcarea A Sovaila S. Sepsis, a 2020 review for the internist. Rom J Intern Med (2020) 58:129–37. doi: 10.2478/rjim-2020-0012 32396142

[24] Danielski LG Giustina AD Bonfante S Barichello T Petronilho F. The NLRP3 inflammasome and its role in sepsis development. Inflammation (2020) 43:24–31. doi: 10.1007/s10753-019-01124-9 31741197

[25] Shi X Tan S Tan S. NLRP3 inflammasome in sepsis (Review). Mol Med Rep (2021) 24:514. doi: 10.3892/mmr.2021.12153 33982766

[26] Busch K Kny M Huang N Klassert TE Stock M Hahn A. Inhibition of the NLRP3/IL-1beta axis protects against sepsis-induced cardiomyopathy. J Cachexia Sarcopenia Muscle (2021) 12:1653–68. doi: 10.1002/jcsm.12763 PMC8718055 34472725

[27] Wu J Yan Z Schwartz DE Yu J Malik AB Hu G. Activation of NLRP3 inflammasome in alveolar macrophages contributes to mechanical stretch-induced lung inflammation and injury. J Immunol (2013) 190:3590–9. doi: 10.4049/jimmunol.1200860 PMC3608749 23436933

[28] Shi CS Shenderov K Huang NN Kabat J Abu-Asab M Fitzgerald KA. Activation of autophagy by inflammatory signals limits IL-1beta production by targeting ubiquitinated inflammasomes for destruction. Nat Immunol (2012) 13:255–63. doi: 10.1038/ni.2215 PMC4116819 22286270

[29] Borges-Rodriguez M Shields CA Travis OK Tramel RW Baik CH Giachelli CA. Platelet inhibition prevents NLRP3 inflammasome activation and sepsis-induced kidney injury. Int J Mol Sci (2021) 22:10330. doi: 10.3390/ijms221910330 34638670 PMC8508664

[30] Li D Ren W Jiang Z Zhu L. Regulation of the NLRP3 inflammasome and macrophage pyroptosis by the p38 MAPK signaling pathway in a mouse model of acute lung injury. Mol Med Rep (2018) 18:4399–409. doi: 10.3892/mmr.2018.9427 PMC6172370 30152849

