# Supplementary material for: Clinical application of serum NLRP3 on the diagnosis and prognosis of sepsis patients complicated with acute respiratory distress syndrome
Source: Front Immunol. 2023 Aug 14;14:1205132. doi: 10.3389/fimmu.2023.1205132 (PMC10462769; doi:10.3389/fimmu.2023.1205132)
Supplement: Supplementary file 1 [file DataSheet_1.pdf]

Supplementary materials

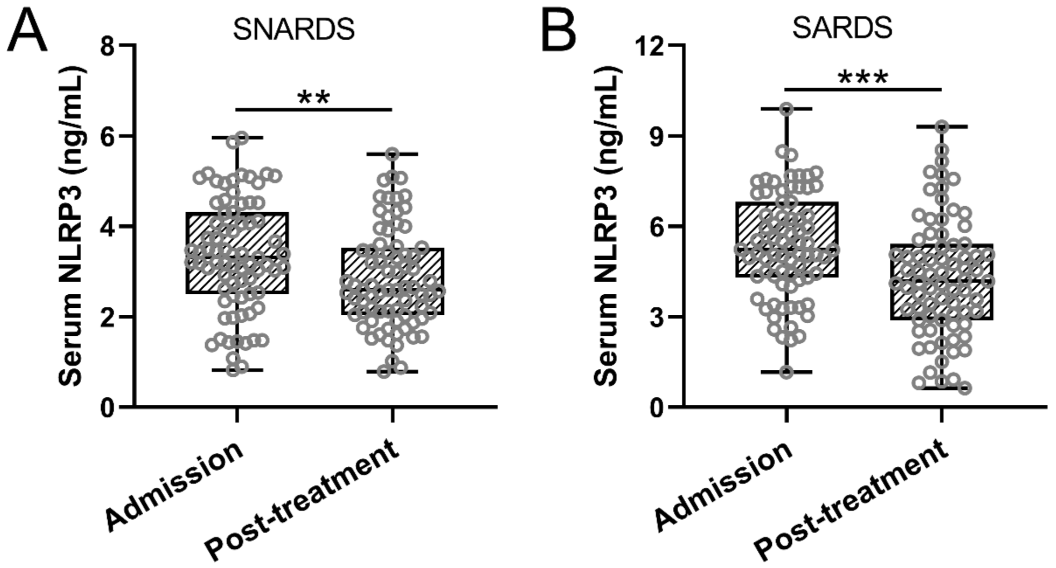

Figure S1. Comparisons of serum NLRP3 at admission and after 7 days of treatment in sepsis patients complicated with ARDS (B) or not (A). **p < 0.01, ***p < 0.001 from Unpaired t test with Welch's correction.

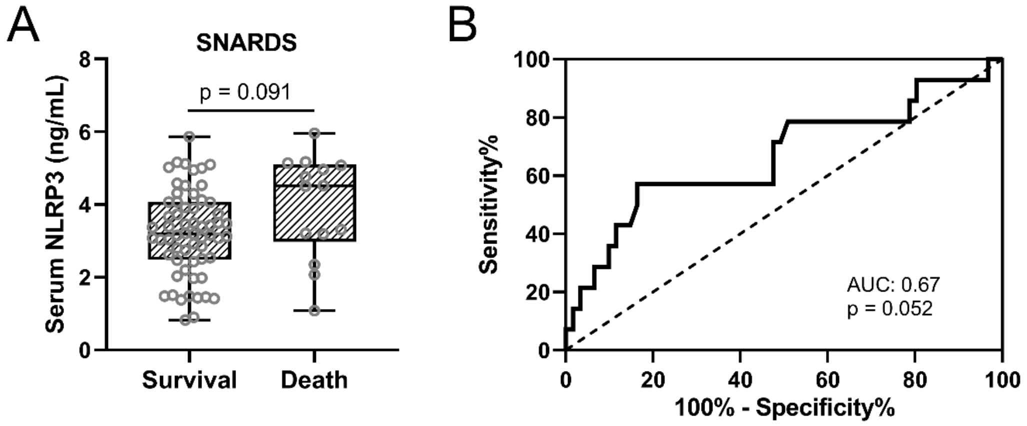

Figure S2. (A), Comparisons of serum NLRP3 between survival (n = 61) and death (n = 14) in sepsis patients not complicated with ARDS after 28 days of follow-up. (B), ROC analysis of predictive values of serum NLRP3 for 28 days of death in sepsis patients not complicated with ARDS.
